# The impact of SGLT2 inhibitors on renal outcomes in patients with type 2 diabetes and chronic kidney disease: systematic review and meta-analysis

**DOI:** 10.3389/fendo.2026.1785822

**Published:** 2026-04-24

**Authors:** Ya Su, Wei Wang, Hui Li

**Affiliations:** Department of Endocrinology, Shaanxi Provincial People’s Hospital, Xi’an, Shaanxi, China

**Keywords:** kidney disease, meta-analysis, renal outcomes, SGLT2 inhibitors, type 2 diabetes

## Abstract

**Objective:**

To analyze and evaluate the impact of SGLT2 inhibitors on the renal outcomes of patients with type 2 diabetes mellitus and chronic kidney disease, and to provide evidence-based basis for clinical rational treatment.

**Methods:**

Relevant literatures on the impact of SGLT2 inhibitors on the renal outcomes of patients with type 2 diabetes mellitus and chronic kidney disease published in domestic and foreign databases were retrieved and collected. The time limit was from the establishment of each database to November 2025. After screening, the quality of the research literature was evaluated using the Cochrane library. Literature management was conducted using NoteExpress 3.2, and data collection and extraction were carried out using Excel 2003. Statistical analysis was performed using RevMan 5.4.1 software. Based on the size of the Q test (P value), it was determined whether there was heterogeneity in the studies, and then the fixed or random effect model was used to calculate the combined effect OR value, and a forest plot was drawn. Then, the publication bias was evaluated by drawing a funnel plot.

**Results:**

A total of 10 studies that met the inclusion criteria were finally included. The meta-analysis results showed that compared with the control group, the eGFR and CrCI levels of patients treated with 5 mg dapagliflozin showed a more significant decline, renal-related adverse events (OR = 0.91, 95% CI: 0.84 to 0.99, P = 0.04), and the occurrence of doubling of serum creatinine, end-stage renal disease, and renal death events (OR = 0.68, 95% CI: 0.60 to 0.78, P < 0.00001). However, there was no statistically significant difference in acute kidney injury or failure between the two groups. Sensitivity analysis suggested that the results of this study were stable and reliable.

**Conclusion:**

SGLT2 inhibitors can cause a short-term decline in eGFR and CrCl, and significantly reduce the risk of renal composite endpoint events. This indicates that their early hemodynamic effects are predictable physiological changes.

**Systematic Review Registration:**

https://www.crd.york.ac.uk/PROSPERO/view/CRD420261294499, identifier CRD420261294499.

## Introduction

Diabetes mellitus (DM) is a major public health challenge currently facing the world. According to data from the International Diabetes Federation, the number of global diabetes patients reached 537 million in 2021, and it is projected to increase to 784 million by 2045 (1). The prevalence of diabetes among adults in China is approximately 10.9%, with the prevalence of type 2 diabetes continuously rising and becoming one of the main causes of end-stage renal disease (ESRD) (2, 3). Diabetic nephropathy is a group of chronic complications mainly characterized by microvascular lesions, and its occurrence and progression are related to chronic hyperglycemia, as well as other risk factors such as obesity, hypertension, dyslipidemia, and genetic susceptibility (4–6). According to statistics, about 27.1% of type 2 diabetes patients in China have chronic kidney disease (7), while in the United States, approximately 40% of adult type 2 diabetes patients have chronic kidney disease (4). Studies have shown that compared with chronic kidney disease caused by glomerulonephritis, patients with type 2 diabetes-related chronic kidney disease have significantly higher hospitalization rates and mortality rates (8, 9). These patients clinically present with persistent albuminuria and/or progressive decline in estimated glomerular filtration rate. As the disease progresses, their risks of cardiovascular events and adverse renal outcomes increase significantly (10). The baseline urine albumin/creatinine ratio and estimated glomerular filtration rate level are important indicators affecting the heart and kidney endpoint events (11, 12). In recent years, with the continuous increase in the number of DM patients in China, the admission rate of DM-related CKD patients has also been increasing, gradually becoming the primary cause of CKD hospitalizations in China (13). This not only severely reduces the quality of life of patients with T2DM and CKD, but also increases the medical burden in China day by day. Therefore, more effective treatments are urgently needed to delay the progression of patients’ conditions.

The sodium-glucose cotransporter 2 inhibitors, as a new type of hypoglycemic drugs, promote the excretion of urine glucose by inhibiting the reabsorption of glucose in the proximal renal tubules (14, 15). Multiple studies have confirmed that they have clear renal protective effects, not only reducing blood sugar, blood pressure, uric acid and proteinuria, but also reducing the risks of cardiovascular events and renal endpoint events (16–18). Since these drugs directly act on the kidneys, the impact of their use on renal function in patients with diabetic nephropathy deserves close attention. Although there are already many qualitative meta-analyses on the renal outcomes of SGLT2 inhibitors, there are still insufficient quantitative comparative studies for the population of type 2 diabetes with chronic kidney disease, and there is a lack of focused discussion on renal outcome indicators. The monitoring and control of early renal function indicators are crucial for preventing severe cardiovascular events and delaying the progression to end-stage renal disease. Therefore, this study intends to conduct a meta-analysis of clinical studies related to SGLT2 inhibitors, systematically evaluating and comparing the renal benefits of the two drugs, in order to provide a basis for rational clinical use.

## Materials and methods

### Study design

This systematic review and meta-analysis was registered in the international prospective register of systematic reviews (PROSPERO) prior to commencement, with the registration number: 2026 CRD420261294499.

### Source of materials and search strategy

The computer retrieves multiple authoritative medical and biomedical literature databases, including PubMed, Embase, Willey Library, and Web of Science. The data is updated up to November 30, 2025.

English search terms.

subject words:”Sodium-Glucose Transporter 2 Inhibitors”, “Sodium Glucose Cotransporter 2 Inhibitor”,”Diabetes Mellitus, “Type 2,”Diabetic Nephropathies,”Renal Insufficiency, “Chronic,Disease Progression,”Glomerular Filtration Rate,”Albuminuria”,”Kidney Failure”, “Chronic”

Free words:”Canagliflozin”, “Dapagliflozin”, “Empagliflozin”, “Ertugliflozin”, “Ipragliflozin”, “Luseogliflozin”, “Tofogliflozin”, “Sotagliflozin”,”T2DM”, “type 2 diabet”, “noninsulin dependent diabet”, “NIDDM”,”Diabetic kidney disease”, “diabetic renal disease”, “chronic kidney disease”, “CKD”, “chronic renal disease”, “renal insufficiency”,”Kidney outcome”, “renal outcome”, “nephropathy progression”, “kidney function”

“eGFR decline”, “estimated glomerular filtration rate”, “albuminuria progression”, “proteinuria”

“end stage renal disease”, ESRD, “end stage kidney disease”, ESKD, “kidney failure”, “renal replacement therapy”, “dialysis”, “renal death”

English search formula:#1 “Sodium-Glucose Transporter 2 Inhibitors”[MeSH] OR (SGLT2 inhibitor* OR SGLT-2 inhibitor* OR canagliflozin OR dapagliflozin OR empagliflozin OR ertugliflozin).

#2 “Diabetes Mellitus, Type 2”[MeSH] OR (T2DM OR “type 2 diabet*” OR NIDDM).

#3 “Diabetic Nephropathies”[MeSH] OR “Renal Insufficiency, Chronic”[MeSH] OR (“diabetic kidney disease” OR CKD OR “chronic kidney disease”).

#4 “Disease Progression”[MeSH] OR “Glomerular Filtration Rate”[MeSH] OR “Albuminuria”[MeSH] OR “Kidney Failure, Chronic”[MeSH] OR (“kidney outcome*” OR “eGFR decline” OR ESRD OR dialysis).

#5 #2 AND #3.

#6 #1 AND #5 AND #4.

### Inclusion and exclusion criteria for the literature

Inclusion criteria: ① Study type: Randomized controlled trial (RCT), regardless of whether blinded method was used; ② Study subjects: Adult patients with T2DM and CKD (UACR ≥ 30mg/g, eGFR ≤ 90ml/min/1.73m²) (aged ≥ 18 years) (19). ③ Intervention measures: Experimental group: SGLT2 inhibitors, without restricting the specific type of SGLT2; Control group: Non-SGLT2 inhibitors. ④ Outcome indicators: Renal-related outcomes; (6) If the same author reports some data repeatedly, the study with the largest sample size or the latest publication will be selected.

Exclusion criteria for the literature: (1) Repetitive, irrelevant studies and review-type literature; (2) Studies without comparison between the experimental group and the control group; (3) Inconsistent outcome indicators of the research; (4) Missing or incomplete data, which cannot be utilized or has obvious errors.

### Literature screening and data extraction

The initial screening was completed by two independent researchers based on the titles and abstracts, and studies that were irrelevant or did not meet the inclusion criteria were excluded. The further screening involved reading the full texts to ensure that the included literature fully met the standards. Disagreements in the literature would be resolved through discussion or third-party arbitration. For the included studies, the two researchers independently conducted data extraction, covering: basic research information, characteristics of the subjects, and outcome indicators, etc. All extracted data were cross-verified to ensure accuracy and consistency. Inconsistencies would be resolved through discussion or expert consultation. During the data extraction process, we systematically recorded and compared the baseline background treatment information of the experimental groups and control groups in each included study, with a particular focus on the usage proportions of angiotensin-converting enzyme inhibitors (ACEI)/angiotensin II receptor antagonists (ARB) (collectively referred to as RAAS inhibitors) and statins.

### Literature quality assessment

Two independent reviewers used the Cochrane risk of bias tool for randomized trials to evaluate the risk of bias of the included studies, covering five areas: randomization process, deviation from the established intervention protocol, missing outcome data, outcome measurement, and selection of reported results. Each area was determined as “low risk”, “unclear”, or “high risk” according to the corresponding algorithm. The two independent reviewers assessed the risk of bias and then conducted a cross-check. The two reviewers discussed any inconsistencies or consulted a third reviewer. Due to the fact that the number of included studies for some outcomes was less than 10, no formal publication bias test (such as funnel plot) was conducted. Since the number of included studies for some of the outcomes was only 10, and no formal publication bias test (such as a funnel plot) was conducted, it was impossible to reliably assess potential publication bias. This ensures that the methodological presentation is more accurate and robust.

### Statistical methods

Literature management was conducted using NoteExpress 3.2 software, and data collection and extraction were carried out using Excel 2003 software. Meta-analysis was performed using Revman 5.4.1 software. The heterogeneity of the extracted data was analyzed using the Q test (P value), and the I2 value was used to evaluate the size of heterogeneity. If P > 0.10 or I2 ≤ 50%, it indicates no heterogeneity, and the fixed effects model (FEM) was used for analysis; otherwise, it indicates the presence of heterogeneity, and the random effects model (REM) was used for analysis. The results of the data synthesis analysis were described using odds ratio (OR) or mean difference (MD) and their 95% confidence intervals (CI), and forest plots were drawn. Sensitivity analysis was used to test the stability of the meta-analysis results, and funnel plots were used to evaluate publication bias. The significance level α = 0.05 (two-sided).

## Result

### Literature search results

**Figure 1:**
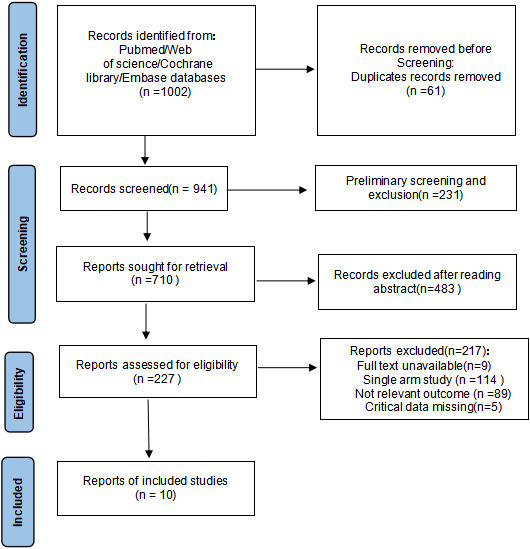
The literature screening process.

Based on the article search strategy, 1002 relevant articles were initially retrieved from databases such as PubMed, Web of Science, Cochrane, and Embase. The duplicate articles from various databases were deleted, and then by reading the titles, abstracts, and full texts of the articles, a total of 10 articles (17, 19–27) were finally included. The literature screening process is shown in **Figure 1**.

### The basic characteristics and quality evaluation of the literature

The baseline data mainly include information such as gender, age, disease duration, treatment plan, and outcome indicators. In the 10 included literature, detailed descriptions of baseline data were provided. The baseline descriptions of both the experimental group and the control group in all the literature were comparable as show in **Table 1**.

**Table 1:** Shows the basic characteristics of the included literature.

Study	Country	Sample	Control Group	Age	Control Group	Sex(Male/Female)	Control Group	Medication	Research type	Inclusion criteria	Baseline medical history
		Experimental group		Experimental group		Experimental group					
Dagogo-Jack S, 2021 (19)	Not mentioned	618/560	598	68.3 ± 7.7/68.2 ± 7.5	68.0 ± 7.5	396/351:222/209	Male:395, Female:203	Ertugliflozin Vs Placebo	RCT	type 2 diabetes	Hypertension, Dyslipidemia, Coronary artery disease, Cerebrovascular disease, Peripheral arterial disease
Kashiwagi A, 2015 (20)	Japan	118	46	63.9 ± 6.59	65.7 ± 6.93	92:26	36/10	Ipragliflozin Vs Placebo	RCT	and albuminuric chronic kidney	Not mentioned
Fioretto P, 2018 (21)	Several countries	160	161	65.3 ± 66.0	66.2 ± 68.0	91:69	91:70	Dapagliflozin Vs Placebo	RCT	type 2 diabetes	Hypertension
Dekkers CCJ, 2018 (22)	Not mentioned	58/93	69	66.0 ± 9.0/66.3 ± 7.4	66.5 ± 7.7	26:32/44:49	29:40	Dapagliflozin Vs Placebo	RCT	and albuminuric chronic kidney	Not mentioned
Wada T(1), 2022 (23)	EA countries	301	303	60.6 ± 9.1	60.9 ± 9.1	214:87	219:84	Canagliflozin Vs Placebo	RCT	type 2 diabetes	Hypertension, Heart failure, CV disease, Coronary, Cerebrovascular, Peripheral vascular, Retinopathy, Neuropathy
Wada T, 2022 (24)	Japan	154	154	62.5 ± 10.5	62.4 ± 11.1	115:39	129:25	Canagliflozin Vs Placebo	RCT	and albuminuric chronic kidney	Hypertension, fracture
Kohan DE, 2014 (25)	Not mentioned	83/85	84	66 ± 8.9/68 ± 7.7	67 ± 8.6	66:17/68:17	53:31	Dapagliflozin Vs Placebo	RCT	type 2 diabetes	Not mentioned
Perkovic V, 2019 (17)	Not mentioned	2202	2199	62.9 ± 9.2	63.2 ± 9.2	1440:762	1467:732	Canagliflozin Vs Placebo	RCT	and albuminuric chronic kidney	Hypertension,Heart failure, Cardiovascular disease
Cherney DZI, 2021 (26)	Multicenter	5499	2747	64.4 ± 8.1	64.4 ± 8.0	3866:1633	1903:844	Ertugliflozin, pooled Vs Placebo	RCT	type 2 diabetes	Not mentioned
Bhatt DL, 2021 (27)	North America, Latin America, Western Europe, Eastern Europe or other regions of the world	5292	5292	69 (63–74)	69 (63–74)	2945:2347	2885:2407	Sotagliflozin Vs Placebo	RCT	and albuminuric chronic kidney	Not mentioned

### Meta-analysis results

**Figure 2:**
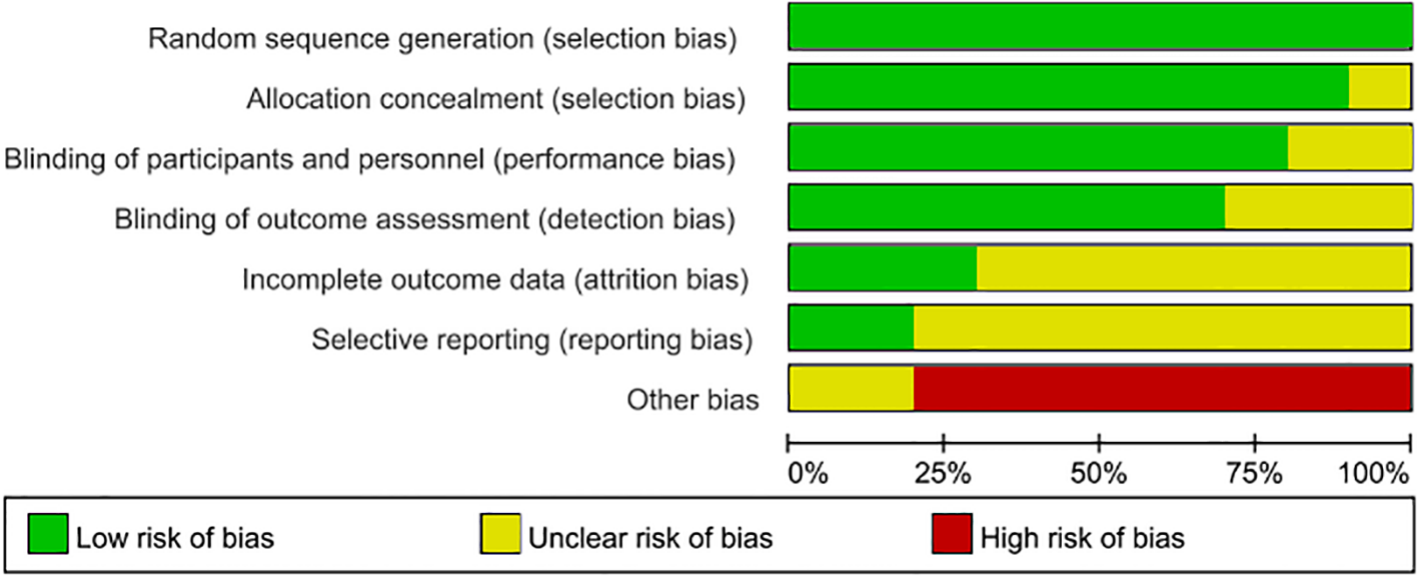
Evaluation of literature bias.

**Figure 3:**
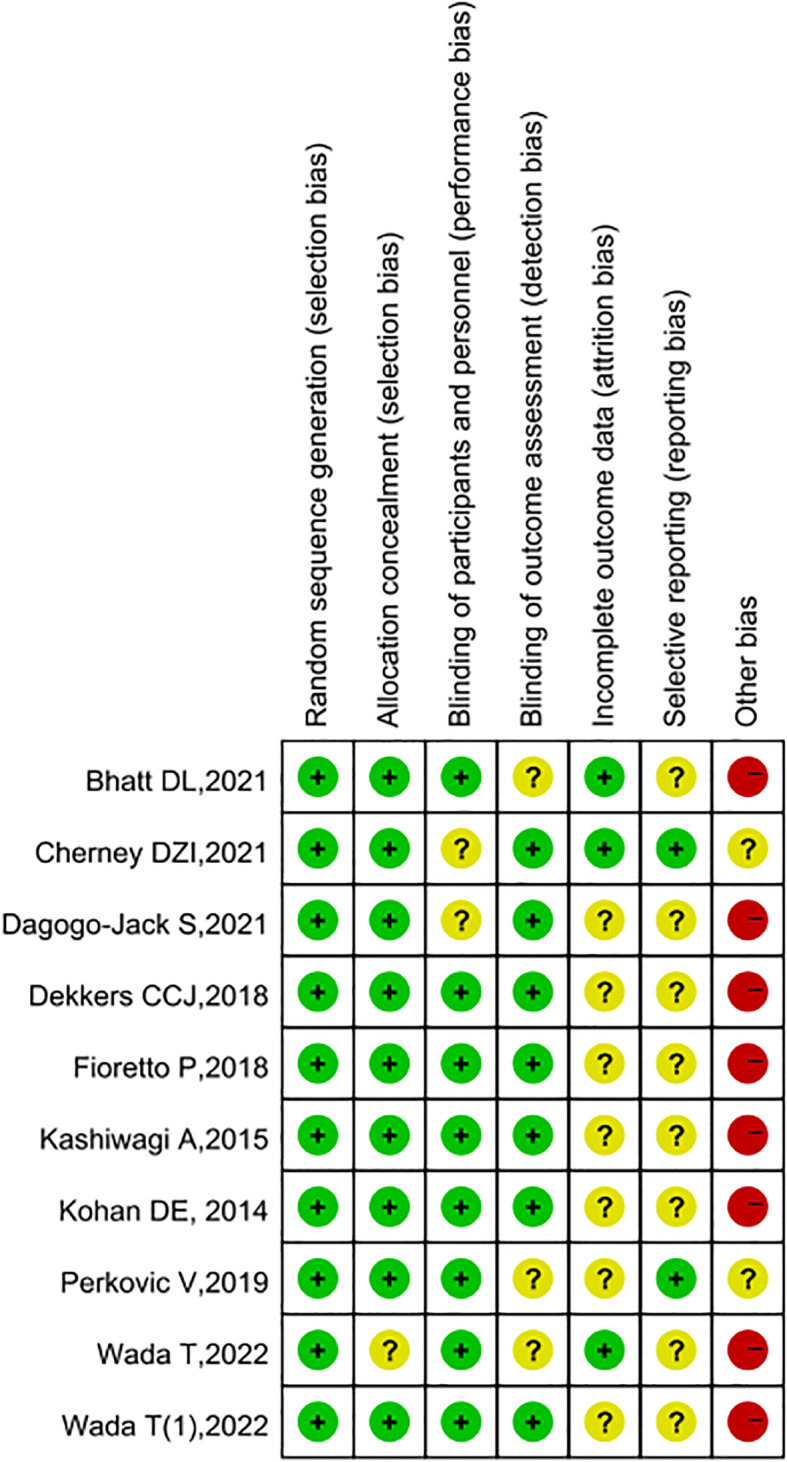
Literature quality evaluation chart.

Two independent reviewers completed the risk of bias assessment and overall quality evaluation of the included RCT studies using the Cochrane risk of bias tool, and the results are presented in **Figure 2** and **Figure 3**. After quality assessment of the included literature, we found that most studies strictly followed the design principles of randomized controlled trials (RCTs). The randomization method was clearly described in most of the literature, ensuring the comparability of the experimental group and the control group at the baseline. However, especially in the double-blind design, it was only applied in a small number of studies; it was basically unclear whether there was selective reporting in the studies, and 8 of the literature did not specify whether there were other biases, but 5 other studies had other biases. Overall, the included literature met our requirements.

### Change in eGFR

**Figure 4:**
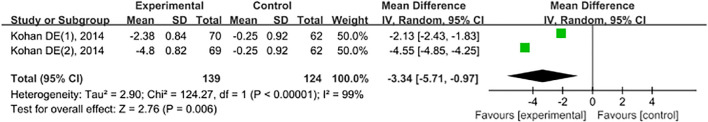
Forest plot of the change values of eGFR in the experimental group and the control group.

Among the included studies, one research (Kohan DE, 2014) reported the effect of SGLT2 inhibitors on the estimated glomerular filtration rate (eGFR). This study included 263 patients with type 2 diabetes and chronic kidney disease. Among them, 139 patients received 5 mg dapagliflozin (experimental group), and 124 patients received 10 mg dapagliflozin (control group). The results showed that compared with the control group, the eGFR levels of patients treated with 5 mg dapagliflozin showed a more significant decline(MD= -3.34,95%CI:-5.71to -0.97, P = 0.006) as shown in **Figure 4**.

### CrCl

**Figure 5:**
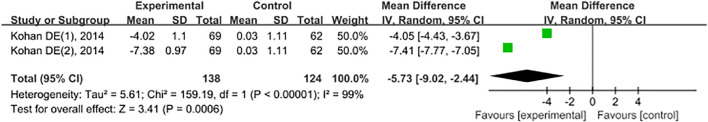
Forest plot comparing CrCl between the experimental group and the control group.

Among the included studies, one research (Kohan DE, 2014) reported the effect of SGLT2 inhibitors on creatinine clearance rate (CrCl). This study included 262 patients with type 2 diabetes and chronic kidney disease. Among them, 138 patients received 5 mg dapagliflozin (experimental group), and 124 patients received 10 mg dapagliflozin (control group). The results showed that compared with the control group, the CrCl levels of patients treated with the low-dose dapagliflozin showed a more significant decrease (MD = -5.73, 95% CI: -9.02 to -2.44, P = 0.0006) as shown in **Figure 5**.

### Adverse events related to renal outcomes

**Figure 6:**
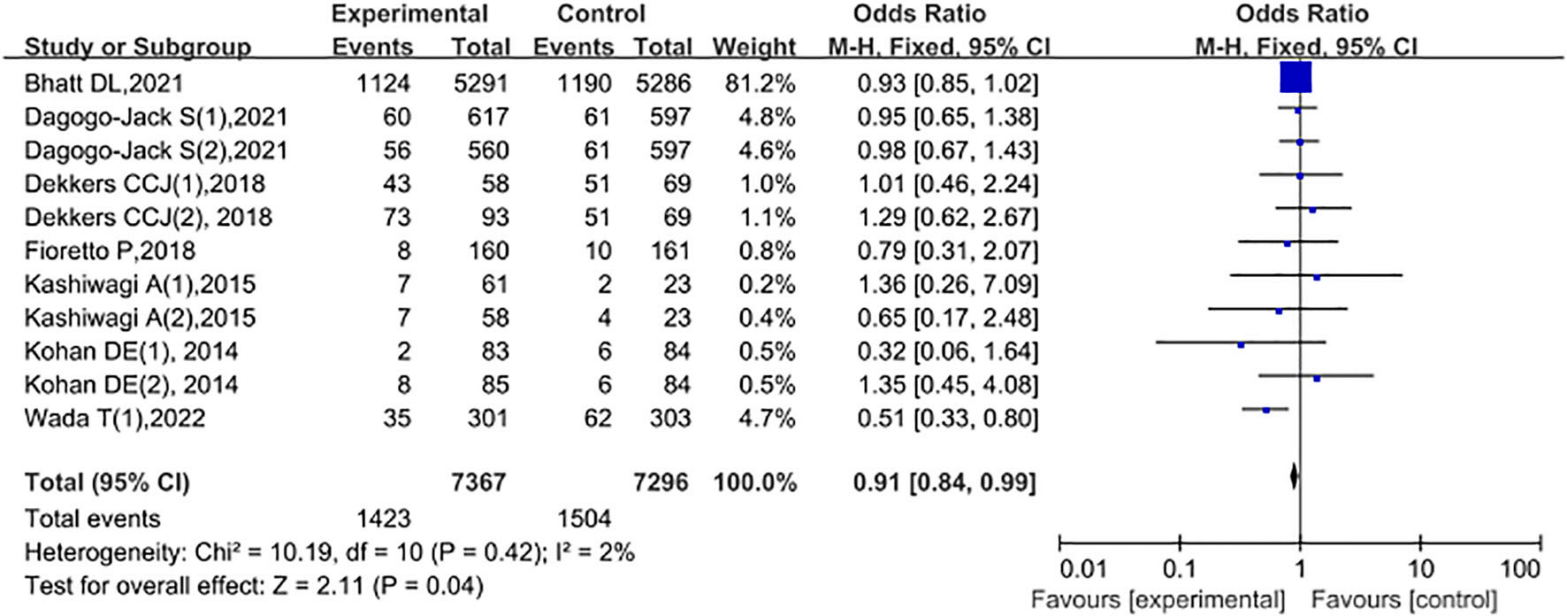
Forest plot comparing adverse events related to renal outcomes between the experimental group and the control group.

A total of 7 studies compared the occurrence of adverse events related to renal outcomes between the experimental group and the control group. Among them, Dagogo-Jack S (1), 2021: the experimental group received Ertugliflozin 5mg; Dagogo-Jack S (2), 2021: the experimental group received Ertugliflozin 10mg; Kohan DE (1), Dekkers CCJ (1), 2018: both the experimental groups received Dapagliflozin 5mg; Kohan DE (2), Dekkers CCJ (2): the experimental groups received Dapagliflozin 10mg; Kashiwagi A (1), 2015: the experimental group had mild renal dysfunction; Kashiwagi A (2), 2015: the experimental group had moderate renal dysfunction. Among them, the experimental group consisted of 7367 cases, and the control group consisted of 7296 cases. The study period was from 2014 to 2022. Heterogeneity tests were conducted on the included studies, and the results showed that P = 0.42 and I2 = 2%, indicating that there was no statistical heterogeneity among different literature studies. Therefore, FEM was used to conduct a meta-analysis on the literature data. The meta-analysis results showed that compared with the control group, the use of SGLT2 inhibitors in patients with type 2 diabetes and chronic kidney disease significantly reduced renal-related adverse events, and the difference was statistically significant (OR = 0.91, 95% CI: 0.84 - 0.99, P = 0.04), as shown in **Figure 6**.

### Acute kidney injury or failure

**Figure 7:**
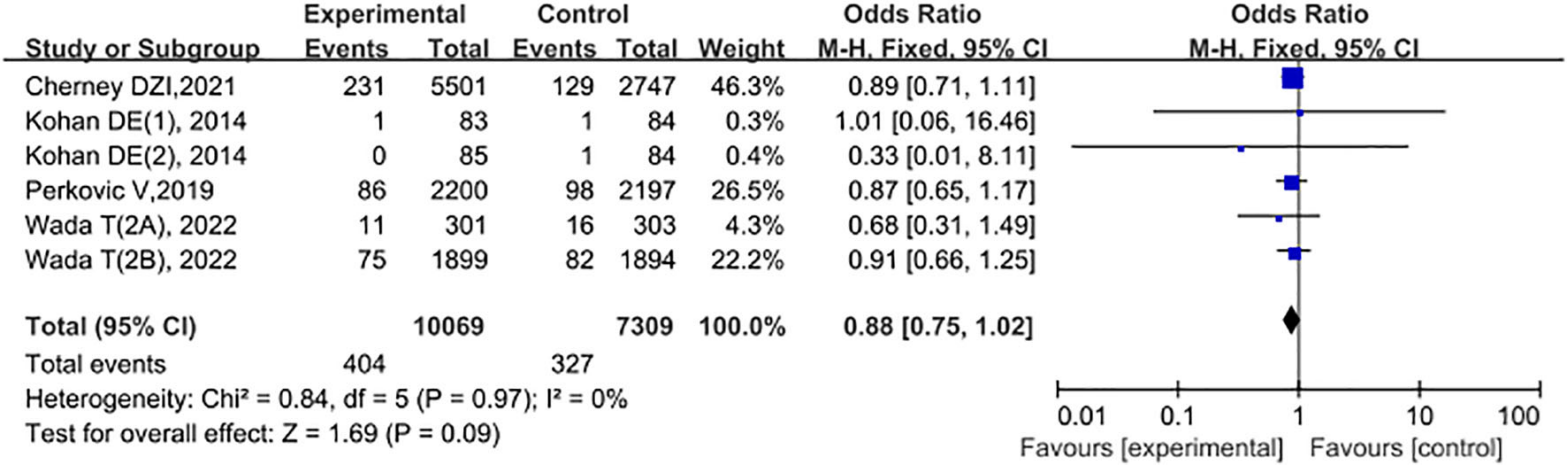
Forest plot comparing acute kidney injury or failure between the experimental group and the control group.

A total of 4 studies compared the acute kidney injury or failure conditions of patients in the experimental group and the control group. Among them, Kohan DE (1), 2014: the experimental group received Dapagliflozin 5 mg; Kohan DE (2), 2014: the experimental group received Dapagliflozin 10 mg; Wada T (2A), 2022: for the East Asian and Southeast Asian populations; Wada T (2B), 2022: for the non-East Asian and Southeast Asian populations. Among them, the experimental group had 10,069 cases, and the control group had 7,309 cases. The study period was from 2019 to 2024. Heterogeneity tests were conducted on the included studies, and the results showed P = 0.97, I2 = 0%, indicating that there was no statistical heterogeneity among different literature studies. Therefore, the FEM was used to conduct a meta-analysis on the literature data. The meta-analysis results showed that there was no statistically significant difference in the occurrence of acute kidney injury or failure between the control group and the experimental group (OR = 0.88, 95% CI: 0.75 - 1.02, P = 0.09), as shown in **Figure 7**.

### Serum creatinine doubles; end-stage renal disease; renal death

**Figure 8:**
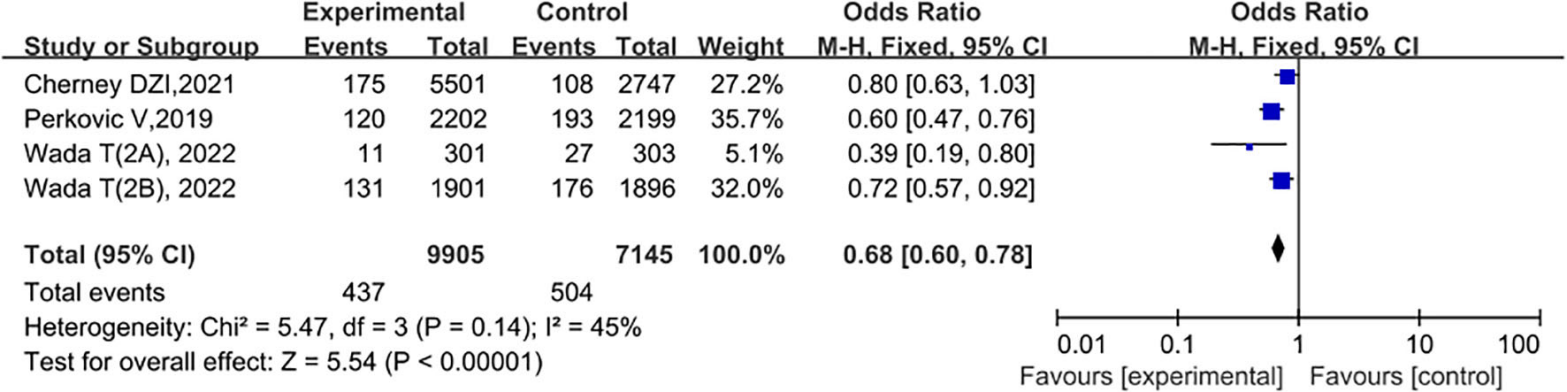
Forest plot showing that serum creatinine doubled in the experimental group compared to the control group; end-stage renal disease; renal death.

A total of 3 studies compared the doubling of serum creatinine, end-stage renal disease, and renal death between the experimental group and the control group. Wada T (2A), 2022 was for the East Asian and Southeast Asian populations, while Wada T (2B), 2022 was for non-East Asian and Southeast Asian populations. Among them, the experimental group consisted of 9,905 cases, and the control group consisted of 7,145 cases. The study period was from 2019 to 2022. Heterogeneity tests were conducted on the included studies, and the results showed P = 0.14 and I2 = 45%, indicating that there was no statistical heterogeneity or a small amount of heterogeneity among different literature studies. Therefore, FEM was used to conduct a meta-analysis on the literature data. The meta-analysis results showed that compared with the control group, the use of SGLT2 inhibitors in patients with type 2 diabetes and chronic kidney disease significantly reduced the events of serum creatinine doubling; end-stage renal disease; and renal death, and the difference was statistically significant (OR = 0.68, 95% CI: 0.60 - 0.78, P < 0.00001), as shown in **Figure 8**.

### Sensitivity analysis

**Figure 9:**
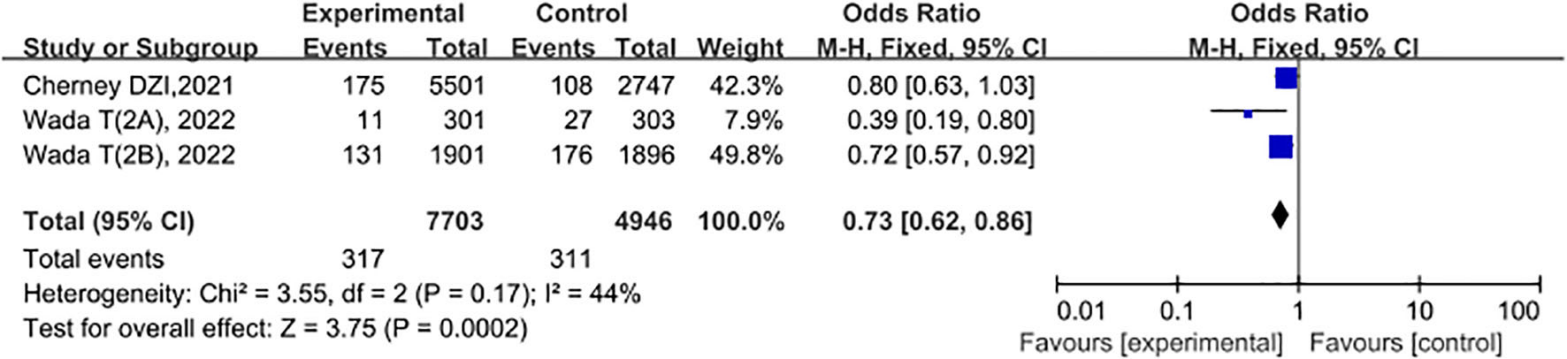
Forest plot of sensitivity analysis.

To assess the stability and reliability of the results of this Meta-analysis, we conducted a sensitivity analysis. By excluding one study at a time and recalculating the combined effect size, we could determine the impact of each study on the overall result. The results showed that when any one study was excluded from the analysis, the direction of the overall effect size did not undergo a significant change, and the range of the confidence interval remained at a relatively stable level, indicating that the model was robust. See **Figure 9**.

### Subgroup analysis

**Figure 10:**
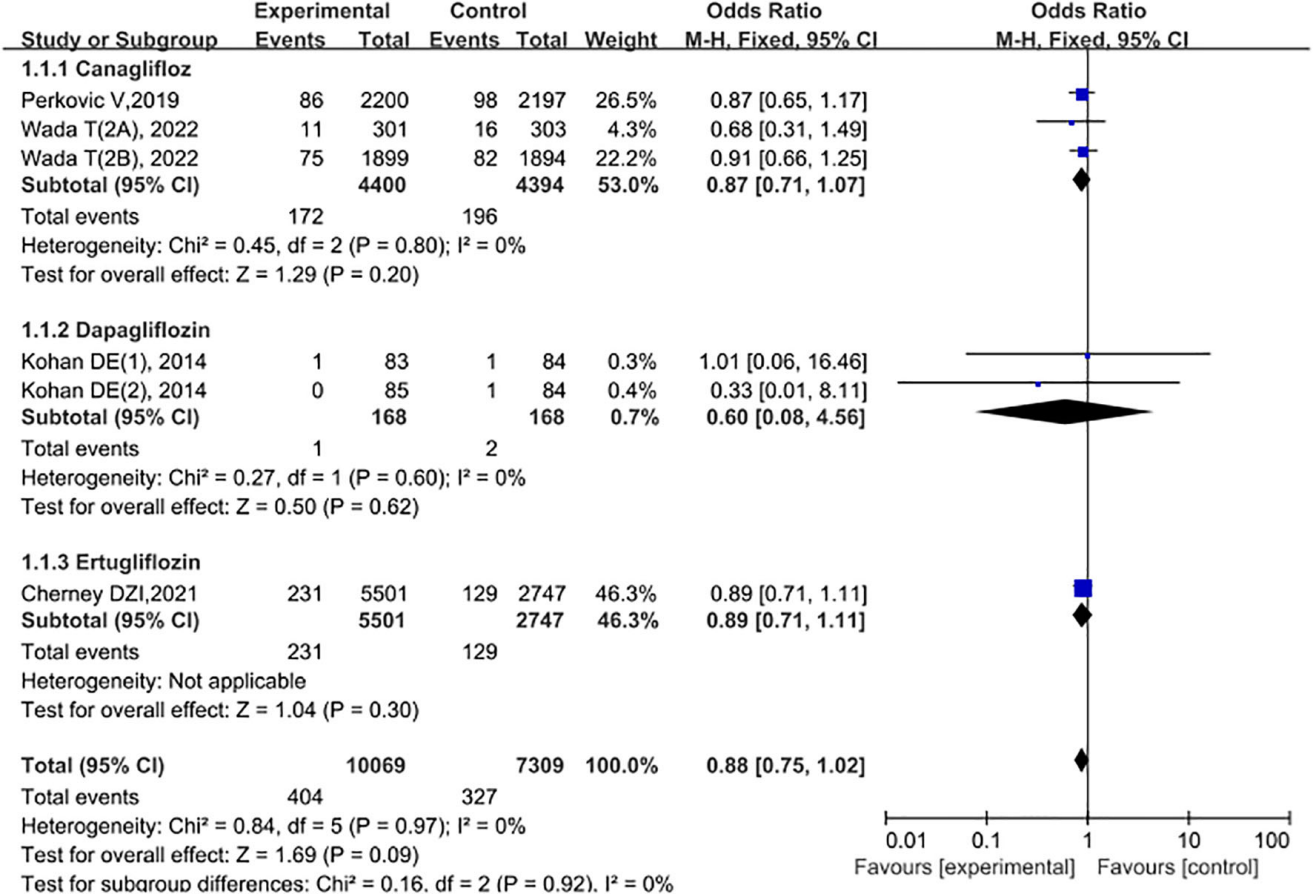
Forest plot of subgroup analysis.

To explore the heterogeneity of this study, we conducted subgroup analysis based on the type of drugs. The results showed that the risk in the SGLT2 inhibitor group was slightly lower than that in the control group (OR = 0.88, 95% CI: 0.75 - 1.02), but did not reach statistical significance (P = 0.09). It is worth noting that there was no significant heterogeneity among the studies (total I² = 0%) See **Figure 10**.

## Discussion

Type 2 diabetes (T2DM) is one of the common chronic diseases in China, and chronic kidney disease (CKD) further increases the health risks and burden of patients (28–30). If T2DM is not effectively controlled, it can lead to damage to multiple organs and blood vessels throughout the body, and become the basis for the occurrence of various complications (5, 31). As a common clinical disease, CKD has led to more than 850 million people worldwide suffering from acute kidney injury (AKI), CKD, and receiving renal replacement therapy (RRT) (32). T2DM combined with CKD not only seriously affects the quality of life of patients, but also significantly increases the risk of end-stage renal disease (ESRD) and adverse cardiovascular events (10, 33). It is worth noting that many patients with progressive CKD often die due to cardiovascular events, even before progressing to chronic renal failure. Therefore, the impact of T2DM combined with CKD is not only limited to individual health, but also constitutes an important public health problem. Although there are currently various treatment methods, these patients still face a high risk of ESRD progression and adverse events. In this context, how to effectively protect the renal function of patients with T2DM combined with CKD, reduce proteinuria, and improve prognosis has become an important direction in medical research.

Sodium-glucose coftransporter 2 (SGLT2) is located on the cell membrane of the proximal tubular epithelial cells of the kidney and works together with glucose transporter (GLUT) to mediate the reabsorption of glucose in the tubular fluid of the kidney (34, 35). In terms of the mechanism of action of SGLT2 inhibitors, they inhibit the reabsorption of glucose and sodium by the proximal tubules, resulting in an increase in sodium ion concentration at the dense plaque, thereby activating the tubule-gluconeurial feedback mechanism, causing the contraction of the afferent arterioles, and ultimately reducing the high filtration and high perfusion state of the glomeruli. Recent translational studies further support the association between this mechanism and the clinical hard endpoint benefits. For example, SGLT2 inhibitor treatment can significantly reduce the levels of markers of renal tubular damage (such as KIM-1, NGAL) in urine, and is associated with the alleviation of podocyte damage and improvement of interstitial fibrosis as shown by renal biopsy, thereby providing crucial pathophysiological evidence for the final transformation of the hemodynamic improvement into long-term kidney protection (15). In addition, these drugs can also reduce the expression of inflammatory factors related to glycation stress and the generation of reactive oxygen species in proximal tubular cells, thereby alleviating oxidative stress, inflammatory response, and renal fibrosis caused by the high sugar state (36, 37). Several recent clinical trials have confirmed that SGLT2 inhibitors not only have the effects of lowering blood sugar, blood pressure, uric acid, and improving glomerular high filtration, but also can reduce the risk of renal and cardiovascular events (16, 38). Therefore, SGLT2 inhibitors provide a new treatment option for patients with T2DM and CKD, but the specific impact on renal function still needs to be further clarified.

In clinical practice, the majority of patients with T2DM and CKD have lipid metabolism disorders, which can promote glomerular arteriosclerosis and accelerate the decline of estimated glomerular filtration rate (eGFR), thereby promoting the progression of kidney disease (39). It is worth noting that the use of SGLT2 inhibitors often leads to a certain degree of eGFR decline in the early stage, suggesting that they may aggravate renal function damage in some cases. In the advanced stage of CKD, the decline in eGFR is more significant (40). This phenomenon is similar to the results of previous studies, and the mechanism may be related to SGLT2 inhibitors causing hypoxia in the glomerular juxtaglomerular apparatus, activating the renin-angiotensin-aldosterone system (RAAS), reducing the reabsorption of albumin in the proximal tubules, and leading to albuminuria, etc. (41, 42). Another animal study has shown that SGLT2 inhibitors may cause collagen deposition, thickening of the glomerular basement membrane, fusion of endothelial cells and foot processes, etc., leading to glomerular damage and mitochondrial dysfunction (43). However, these conclusions are mostly based on animal studies, and clinical data are still limited. Moreover, the sample sizes included in related studies are insufficient, so the impact of SGLT2 inhibitors on renal function still requires more high-quality evidence to support.

This study shows that these drugs can reduce CrCI at an early stage of use and present a trend of delaying the decline of eGFR. This result is consistent with previous studies (17). The main reason is that SGLT2 inhibitor drugs share a common pharmacological mechanism, that is, by inhibiting the reabsorption of glucose and sodium in the proximal tubules of the kidneys, increasing the transport of sodium ions from the lumen to the dense plaque, thereby activating the tubuloglomerular feedback mechanism, causing the contraction of the afferent arterioles, reducing the intraglomerular pressure and filtration load. This acute hemodynamic effect is manifested as a temporary decrease in estimated glomerular filtration rate (eGFR) or a decrease in creatinine clearance rate (CrCl) in the early stage of use, while in the long term, it reduces the high filtration and high perfusion state of the glomerulus, reduces proteinuria and tubulointerstitial damage, and ultimately plays a role in delaying the decline of renal function, which is completely consistent with the trend of a small decrease in eGFR and a slower long-term slope observed in several large randomized controlled trials (such as CREDENCE, DAPA-CKD, etc.). This confirms the reproducibility and cross-study consistency of the characteristic pattern of the impact of these drugs on renal function (44).

In terms of safety, SGLT2 inhibitors, as a class of drugs with a relatively balanced effect distributed throughout the cardiovascular and renal systems, mainly target the heart and blood vessels. They exert cardiorenal protective effects by reducing oxidative stress, improving endothelial function, inhibiting myocardial hypertrophy, and lowering plasma brain natriuretic peptide levels (45). The FIDELIO-DKD trial analysis indicated that finerenone could reduce the risks of composite cardiovascular endpoints and composite renal endpoints in the early stage of treatment (43). This study also confirmed that SGLT2 inhibitors can significantly reduce the occurrence of serum creatinine doubling, ESRD, and death due to kidney-related causes in patients with T2DM and CKD.

This study has certain limitations: Although a relatively systematic search strategy has been adopted to cover major databases, there may still be studies that met the criteria but were not included; all foreign literature is limited to English publications, which may introduce language bias. Additionally, the sample sizes of the included studies vary, and there is significant heterogeneity in aspects such as drug dosage and combination therapy in the intervention measures, as well as differences in follow-up duration. Only 2–3 randomized controlled trials (RCTs) of various SGLT2 inhibitors were included, and the number of studies is limited, which may increase the risk of bias. The efficacy of these drugs in the advanced stage of chronic kidney disease (CKD) or in populations with different genetic backgrounds remains an important research topic for the future. Therefore, in the future, larger sample, multi-center, and well-designed RCTs are still needed to scientifically group the intervention measures and conduct long-term follow-up, thereby providing more reliable evidence-based medical evidence for the treatment of T2DM with SGLT2 inhibitors in combination with CKD.

The results of this study indicate that in the population with type 2 diabetes and chronic kidney disease, SGLT2 inhibitors (represented by dapagliflozin) can significantly reduce the risk of the composite hard endpoint consisting of serum creatinine doubling, end-stage renal disease, and renal death. This core finding is not isolated but rather highly consistent with the high-level clinical evidence that has been continuously accumulating over the years and covers a wider range of patient populations. Together, they have jointly depicted the clear cardiorenal protection profile of SGLT2 inhibitors. For instance, a recent meta-analysis on patients with type 2 diabetes who underwent percutaneous coronary intervention (PCI) showed that compared with DPP-4 inhibitors, SGLT2 inhibitors significantly reduced all-cause mortality, slowed down the deterioration of renal function, and decreased the risk of hospitalization for heart failure (46). This study further verified the organ-protective effects beyond hypoglycemic action of SGLT2 inhibitors in a specific population with a high cardiovascular background. The results of our study—namely, observing a 28% reduction in the risk of renal hard endpoints (OR = 0.68)—are in strong agreement with the above evidence. Notably, the reduction in this composite renal endpoint directly translates into meaningful patient-centered outcomes. Large-scale long-term trials (e.g., CREDENCE, DAPA-CKD) have demonstrated that such reductions correspond to an approximately 30%–40% lower risk of end-stage renal disease (ESRD) and significantly fewer hospitalizations for renal or heart failure causes. Therefore, our findings reinforce that the benefits observed in surrogate markers and composite endpoints ultimately lead to delayed disease progression, reduced major clinical events, and improved long-term prognosis for patients.

Although this study and the above PCI postoperative analysis differ in specific populations and control settings, they jointly point to a conclusion: SGLT2 inhibitors have become a cornerstone drug that can provide extensive clinical benefits throughout the cardiovascular and renal systems for patients with type 2 diabetes. The early acute decline in eGFR observed in our analysis, likely reflecting hemodynamic adjustment, may initiate the attenuation of long-term GFR decline slope—a key mechanism that delays the need for renal replacement therapy such as dialysis. However, it should be acknowledged that high-quality long-term evidence directly linking SGLT2 inhibitor use to further patient-centered outcomes, such as health-related quality of life or healthcare economic burden, remains relatively limited and warrants future real-world studies or trials incorporating patient-reported outcomes.

Therefore, our research results further support the idea that for eligible patients with type 2 diabetes and chronic kidney disease, regardless of whether they have clear atherosclerotic cardiovascular disease, SGLT2 inhibitors should be actively considered as an important component of the comprehensive management strategy.

In conclusion, based on the available evidence, SGLT2 inhibitors demonstrate good efficacy and safety in the treatment of T2DM patients with CKD. During clinical application, it is necessary to closely monitor the patient’s renal function and adjust the medication regimen according to specific circumstances. Due to the lack of sufficient high-quality RCT evidence at present, a cautious attitude should be maintained towards the existing conclusions. Further verification is needed.

## Data Availability

The raw data supporting the conclusions of this article will be made available by the authors, without undue reservation.
